# Expansion of CD25-Negative Forkhead Box P3-Positive T Cells during HIV and *Mycobacterium tuberculosis* Infection

**DOI:** 10.3389/fimmu.2017.00528

**Published:** 2017-05-09

**Authors:** Matías T. Angerami, Guadalupe V. Suarez, María B. Vecchione, Natalia Laufer, Diego Ameri, Graciela Ben, Hector Perez, Omar Sued, Horacio Salomón, María F. Quiroga

**Affiliations:** ^1^Instituto de Investigaciones Biomédicas en Retrovirus y Sida (INBIRS), Facultad de Medicina, Universidad de Buenos Aires, Consejo Nacional de Investigaciones Científicas y Técnicas (CONICET), Buenos Aires, Argentina; ^2^Hospital Juan A. Fernández, Buenos Aires, Argentina; ^3^Área de Investigaciones Médicas, Fundación Huésped, Buenos Aires, Argentina

**Keywords:** human, infectious immunity bacteria, regulatory mechanisms, cytokines, effector/memory Treg populations

## Abstract

Tuberculosis (TB) and HIV alter the immune system, and coinfected (HIV-TB) individuals usually present deregulations of T-lymphocytic immune response. We previously observed an increased frequency of “unconventional” CD4^+^CD25^−^FoxP3^+^ Treg (uTreg) population during HIV-TB disease. Therefore, we aimed to explore the phenotype and function of uTreg and conventional CD4^+^CD25^+^FoxP3^+^ Treg subsets (cTreg) in this context. We evaluated the expression of CD39, programmed cell death protein 1 (PD1), glucocorticoid-induced tumor necrosis factor receptor (GITR), and the effector/memory distribution by flow cytometry in cTreg and uTreg. Also, IL-10, TGF-β, IFN-γ production, and the suppressor capacity of uTregs were analyzed in cocultures with effector lymphocytes and compared with the effect of regulatory T cells (Tregs). We found diminished expression of CD39 and higher levels of PD1 on uTreg compared to cTreg in both HIV-TB and healthy donors (HD). In addition, uTreg and cTreg showed differences in maturation status in both HIV-TB and HD groups, due to the expansion of effector memory uTregs. Interestingly, both HIV-TB and HD showed a pronounced production of IFN-γ in uTreg population, though no significant differences were observed for IL-10 and TGF-β production between uTreg and cTreg. Moreover, IFN-γ^+^ cells were restricted to the CD39^−^ uTreg population. Finally, when the suppressor capacity was evaluated, both uTreg and cTreg inhibited polyclonal T cell-proliferation and IFN-γ production in a similar extent. These findings suggest that uTregs, which are expanded during HIV-TB coinfection, exert regulatory functions in a similar way to cTregs despite an altered surface expression of Treg characteristic markers and differences in cytokine production.

## Introduction

Tuberculosis (TB) remains to be a serious threat, especially in HIV patients. In fact, *Mycobacterium tuberculosis* (*Mtb*) is the most common cause of AIDS-related deaths, and HIV is the most powerful known risk factor predisposing for TB infection and progression to active disease (1). Both pathogens synergistically alter the immune system and accelerate the deterioration of immunological functions. Besides the risk for the infected individuals, HIV-TB coinfection leads to a huge burden over health-care systems, especially in resource-limited countries (2).

It is well known that regulatory T cells (Tregs) play a central role in the induction and maintenance of tolerance mechanisms. Deficiencies in this cell population can lead to autoimmune processes or to a poor response to pathogens (3). This Treg subset is characterized by high expression of CD25 (the alpha chain of the IL-2 receptor), but until now the most specific marker for this population is the forkhead box P3 (FoxP3) transcription factor, whose expression is critical for a proper suppressor function. Accordingly, the best known suppressor T cell population is defined as CD4^+^CD25^+^FoxP3^+^, or “conventional” Tregs (cTreg). Nevertheless, Tregs engulf several subpopulations with different functional capacities (4). Besides, an enhanced expression of FoxP3 in human activated T lymphocytes has been observed without conferring any regulatory properties to this cell population (5).

HIV-TB coinfection sets up a complex scenario, where the immune activation induced by both pathogens accelerates a decline in immune functions, leading to death if untreated (2). In this context, the expansion of FoxP3^+^ Tregs is a discouraging feature, as this leads to the suppression of antigen-specific immune responses, including those specific for *M. tuberculosis* (6). We previously reported a unique population of CD4^+^CD25^−^FoxP3^+^ T lymphocytes that was expanded in a cohort of HIV-TB coinfected patients, a population we defined as “unconventional” Tregs (uTreg) (7). This unique subset was also described in the context of systemic lupus erythematosus (SLE), but not in other autoimmune disorders, such as rheumatoid arthritis (8). Moreover, the nature of these uTreg lymphocytes is controversial, since some authors propose that these cells encompass immature regulatory cells, while others suggest that uTregs are effector CD4^+^ T cells with a transient expression of FoxP3 because of T cell activation (9).

The role of Tregs in TB has been widely studied over the past years, yet some gaps need further research. In this scenario, Treg heterogeneity has not been deeply investigated, although previous studies indicate the existence of functionally diverse populations (10). Remarkably, surface and intracellular expression of certain molecules also determine cell functions. For instance, CD39 expression will define a more pronounced inhibitory profile for Tregs, determining the shape of the immune response to *Mtb* infection (11). Additionally, programmed cell death protein 1 (PD1) expression will also define Treg function and exhaustion as described in TB patients (12). Finally, the memory/effector characterization might define T cell memory pools, which exert different suppressor capacities among distinct Treg subpopulations. For instance, Mailloux et al. have demonstrated that peripheral effector memory (EM) Tregs in healthy donors (HD) constitute only 2% of all Tregs but when isolated they exhibit greater *in vitro* suppressor activity compared to central memory (CM) or naïve (N) cells (13).

Thus, we aimed to explore the nature of “unconventional” Tregs, unraveling their phenotype and inhibitory capacity to get a better understanding of the pathogenic mechanisms involved in *M. tuberculosis* immune response in the context of HIV infection. We could observe some unique features in these uTregs, but still they maintained their inhibitory capacity as they suppressed both proliferation and cytokine production from effector T cells in an *in vitro* setting.

## Materials and Methods

### Individuals Enrolled

The present cross-sectional study recruited the following population (Table 1): 1. HIV-1 infected patients with active TB (HIV-TB) who received anti-TB therapy for less than 1 week, 2. Asymptomatic HIV-1 infected patients (HIV^+^) as determined by ELISA and confirmatory Western Blot who were tuberculin skin test negative (TSTneg), 3. HIV-1 infected individuals latently infected with *Mtb* (HIV-LTB), 4. HD TSTneg. All individuals were Bacillus Calmette–Guérin vaccinated and evaluated at Hospital J. A. Fernandez, Buenos Aires. Some HIV-1 infected individuals were under antiretroviral treatment following local and international guidelines. None of the recruited individuals presented associated infections, neither autoimmune nor endocrine imbalance. The Ethics Committees from Fundación Huésped and from the University of Buenos Aires School of Medicine approved the current study. In addition, written informed consent was documented from all study subjects.

**Table 1:** **Epidemiological characteristics of the subjects enrolled**.

	HIV-TB	HIV-LTB	HIV	Healthy donors
Number of subjects enrolled	28	18	17	19
Median age in years (IQR)	39 (33–44)	35 (29–40)	35 (28–47)	37 (29–43)
Female/male distribution	10/18	8/10	6/11	12/17
HIV status	+	+	+	−
Median CD4 count (IQR)	129 (81–271)	386 (297–575)	307 (162–523)	834 (570–12,923)
Median viral load (IQR)	28,771 (89–93,136)	10,195 (124–16,742)	12,934 (1,055–21,400)	N/A

*IQR, interquartile range*.

### Cell Preparations and Culture Conditions

PBMCs were isolated by density gradient centrifugation on Ficoll–Hypaque and cultured at 2 × 10^6^/ml with PMA and ionomycin (5 and 500 ng/ml, respectively, Sigma-Aldrich) and brefeldin A (3 µg/ml, Sigma-Aldrich) in RPMI 1640 medium (Sigma-Aldrich) supplemented with 10% fetal calf serum (PAA), 2 mM l-glutamine (Gibco BRL), 100 U/ml penicillin (Gibco BRL), and 100 µg/ml streptomycin (Gibco BRL) at 37°C for 6 h. Then, cells were washed, stained for surface and intracellular markers, and analyzed by flow cytometry (see below).

### Flow Cytometry

PBMCs (1 × 10^6^) were stained with CD4 PerCP, CD25 Alexa488, and FoxP3 PE for the identification of Treg populations (BD FoxP3 staining protocol, according to the manufacturer’s instructions). CD4 PerCP, CD25 Alexa488, CD27 APC, and CD45RA PE-Cy7 (eBioscience) were used to assess surface phenotype. In some experiments, CD39 APC, PD1 PE-Cy7, and glucocorticoid-induced tumor necrosis factor receptor (GITR) APC (Biolegend) were analyzed on Treg populations. Intracellular cytokine staining was performed to determine IFN-γ and IL-10 production at the single-cell level as described previously using anti-human IFN-γ-PE-Cy7 and anti-human IL-10 APC (both from Biolegend). For the evaluation of TGF-β secretion, the surface expression of the latency-associated peptide (LAP) protein (anti-human LAP-PE-Cy7, Biolegend) was assessed. For all flow cytometry experiments, sample acquisition and analysis were carried out on a FACSCanto flow cytometer using the BD FACSDiva software (BD Biosciences). Negative control samples were incubated with irrelevant, isotype-matched mAbs in parallel with experimental samples.

### Functional Assays

#### Treg Isolation

Due to the limitations of FoxP3 to separate human Tregs for functional studies, we first developed a phenotyping strategy based on the expression of CD39 surface marker as a surrogate of FoxP3 for subsequent FACS sorting experiments. This strategy allowed us to obtain three specific populations: CD4^+^C25^+^CD39^+^ (cTreg), CD4^+^CD25^−^CD39^+^ (uTreg), and CD4^+^CD25^−^CD39^−^ [effector (Eff)]. These T cell subpopulations were isolated from HD samples by flow cytometry cell sorting on a FACSAria (BD Biosciences). PBMCs were stained with mAb against CD4 PerCP, CD25 Alexa 488, and CD39 APC for 20 min at 4°C. Sort gates were additionally restricted to a lymphocyte gate as determined by typical forward and side scatter characteristics. Then, CD4^+^CD25^+^CD39^+^ (cTreg), CD4^+^CD25^−^CD39^+^ (uTreg), and CD4^+^CD25^−^CD39^−^ (Eff) T cells were enriched. In parallel, samples were labeled for CD4, CD25, CD39, and FoxP3 to determine the percentage of FoxP3-positive cells in the sorted subpopulation.

#### Suppression Assays

For the assessment of maximum T cell proliferation, FACS-sorted CD4^+^CD25^−^CD39^−^ (effector T cells) from HD were stimulated in U-bottom 96-well plates with soluble anti-CD3 mAb (10 ng/ml) and allogenic PBMC (1 × 10^5^) pretreated with mitomicin (25 µg/ml, Sigma-Aldrich) as feeder cells. Additionally, suppression was determined by coculturing 2.5 × 10^4^ CD4^+^CD25^−^CD39^−^ (Eff) T cells in the presence of FACS-sorted CD4^+^CD25^−^CD39^+^ (uTregs) or CD4^+^CD25^+^CD39^+^ (cTregs) T cells at 1:1, 1:2, 1:4, 1:8, 1:16, 1:32 Treg:Eff ratios in RPMI 1640 plus 10% FCS at 37°C in a humidified CO_2_ containing atmosphere. T cell proliferation was assessed at day 5 by measuring [methyl-3H] thymidine (1 μCi per well, GE Healthcare) incorporation. The proliferative response of stimulated CD4^+^CD25^−^CD39^−^ effector T cells in the absence of CD4^+^CD25^−^CD39^+^ or CD4^+^CD25^+^CD39^+^ Tregs was defined as 100%. Background proliferation was determined from cultures of unstimulated CD4^+^CD25^−^CD39^−^ effector T cells. Cell-free supernatants were collected on day 4 of culture previous to [methyl-3H] thymidine addition and analyzed for cytokine concentrations by ELISA.

### Statistical Analysis

Statistical analyses were conducted using GraphPad Prism 5 version 5.04. Comparisons between two groups were evaluated by the Wilcoxon test or Mann–Whitney test for paired or unpaired samples, respectively. Comparisons between three or more groups were done using the Kruskal–Wallis analysis of variance followed by *post hoc* comparisons (Dunns), when applicable. For paired samples, Friedmand test was performed. Correlations were determined using the Spearman’s rank test. Statistical analysis and display of multicomponent distributions were performed by partial permutation test using SPICE v5.1 (http://exon.niaid.nih.gov/spice/) (14). For all comparisons, a *p* value <0.05 was considered significant.

## Results

### Expansion of Unconventional CD25-Negative Treg Population in HIV-TB Patients

**Figure 1:**
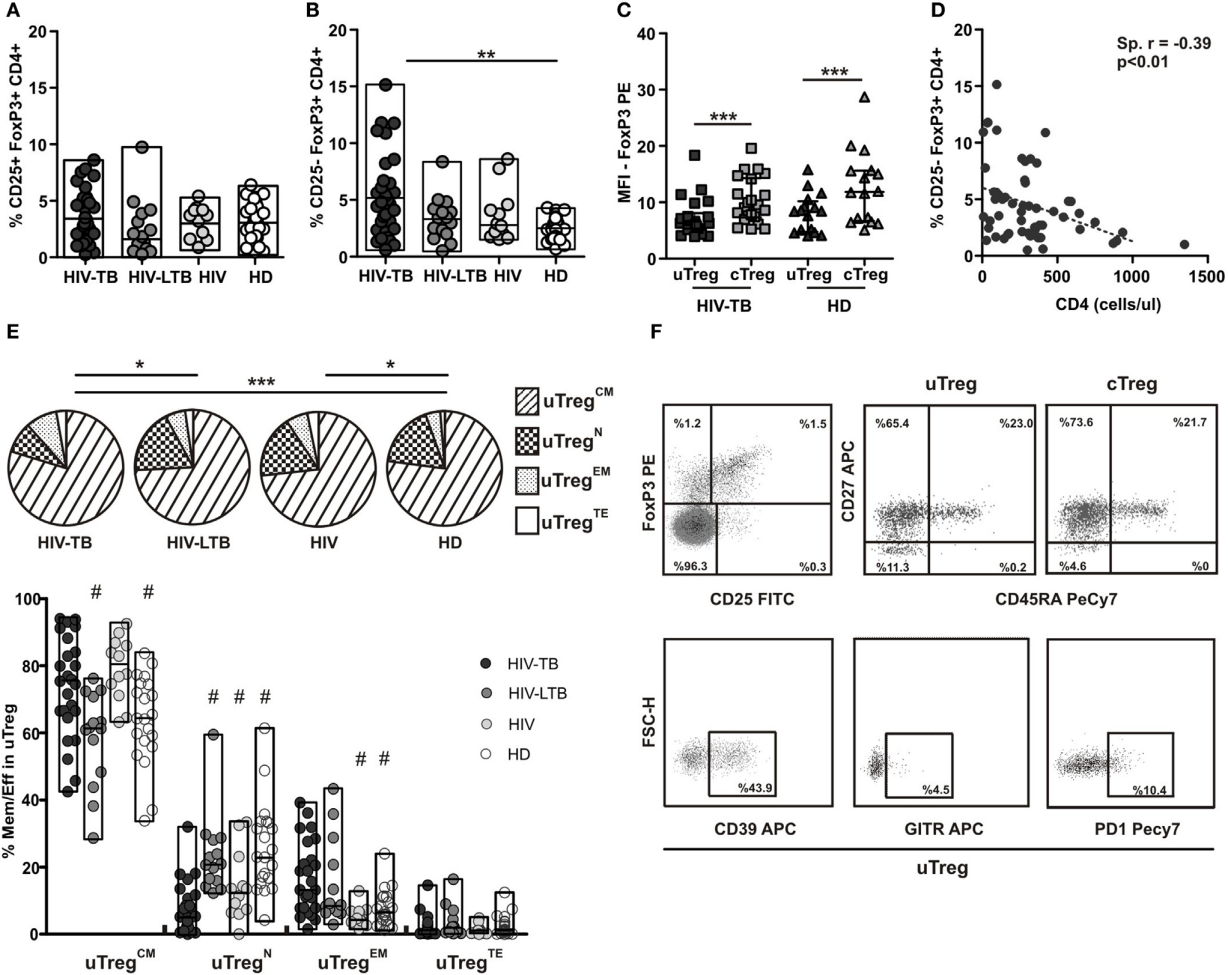
**Analysis of cTreg and uTreg populations across the spectrum of tuberculosis (TB) infection**. PBMC from HIV-TB, HIV-LTB, HIV^+^, and healthy donors (HD) individuals were stained for cell surface expression of CD4, CD25, CD39, glucocorticoid-induced tumor necrosis factor receptor (GITR), programmed cell death protein 1 (PD1), CD45RA, CD27, and intracellular FoxP3 and analyzed by flow cytometry. Cells were gated based first on the basis of CD4 expression, then CD4^+^ cells were analyzed for the expression of CD25 and FoxP3 in order to define “conventional” and “unconventional” regulatory T cells (Tregs), and finally the expression of CD45RA/CD27 was determined in uTregs and cTregs. **(A)** Evaluation of the percentage of cTreg population in HIV-TB, HIV-LTB, HIV^+^, and HD. **(B)** Comparison of the percentage of uTreg between groups. **(C)** Mean fluorescence intensity (MFI) of FoxP3 was measured to compare the level of expression of this molecule in uTreg and cTreg of HIV-TB and HD. Wilcoxon test was used for statistical comparison between the two different subsets. **(D)** Correlation analysis between the absolute number of CD4^+^ T cells and the percentage of uTreg from HIV-infected patients (HIV-TB, HIV-LTB, and HIV). **(E)** The expression of CD27 and CD45RA on CD4^+^ T cells from HIV-TB, HIV-LTB, HIV^+^, and HD patients was analyzed by flow cytometry; (top) pie charts summarize the data and each slice corresponds to the mean proportion of uTreg cells for each phenotype. (Bottom) Possible phenotypes are shown on the *x*-axis whereas percentages of distinct T-cell subsets within uTreg cells are shown on the *y*-axis. **(F)** Representative dot plots of Treg identification, maturation status distribution and surface CD39, PD1, and GITR expression. For the comparison between the four groups, Kruskal–Wallis test was performed followed by Dunns posttest. Horizontal lines represent the median range and each point represents an individual subject; asterisks indicate a significant difference between groups; *p < 0.05; **p < 0.01; ***p < 0.001. Spearman test was performed to evaluate correlations between variables. Comparisons of phenotype distribution were performed using the partial permutation test as described in Ref. (14) and the Kruskal–Wallis test followed by Dunn’s multiple comparisons posttest.

We previously showed that HIV-TB patients present an increased frequency of an “unconventional” regulatory T cell population (uTreg), defined as CD25^−^FoxP3^+^CD4^+^ T cells (7). This unique population was first described in the context of SLE (8, 9, 15). Therefore, we performed a detailed phenotypic analysis of this particular population in a large number of patients. In contrast to “conventional” CD25^+^FoxP3^+^CD4^+^ Treg (cTreg), which exhibited similar frequencies in HIV-TB coinfected patients, HIV^+^ individuals latently infected with TB (HIV-TBL), HIV^+^ persons, and HD (Figure 1A), uTreg frequencies were significantly higher in HIV-TB than HD (Figure 1B) similar to our previous results (7). Even though we did not observe any significant differences between HIV-TB and HIV-TBL or HIV, the last two groups showed similar frequencies to those seen in the HD group (Figure 1B). Additionally, we observed a diminished expression of FoxP3 per cell—in terms of mean fluorescence intensity—in uTreg compared to cTreg in both HIV-TB and HD individuals (*p* < 0.01, Figure 1C), suggesting a reduced suppressor capacity in uTreg compared with cTreg, as described previously (9). Moreover, since the absolute number of CD4^+^ T cells of infected patients negatively correlated with the percentage of uTreg, the expansion of this cell subset could be related with the immune status of the patient (Figure 1D).

Suppressor Treg capacity may rely on specific TCR-dependent activation (16), suggesting that Tregs, similar to conventional effector T cells, modify their phenotype as they activate or expand. To explore this issue, peripheral uTregs were phenotypically discriminated in terms of CD27/CD45RA expression by flow cytometry as naïve (Treg^N^), central memory (Treg^CM^), effector memory (Treg^EM^), and terminal effector (Treg^TE^) Tregs as described elsewhere (13). Thus, partial permutation test (14) evinced dissimilar memory/effector distribution when comparing HIV-TB to HD or HIV-LTB individuals (Figure 1E, pies and Figure 1F). These differences where due to augmented uTreg^CM^ proportions in HIV-TB patients compared to HIV-LTB and HD, diminished percentages of uTreg^N^ among HIV-TB versus HIV-LTB and HD, and also augmented proportions of HIV-TB uTreg^EM^ compared to both HIV^+^ and HD persons (Figure 1E, bars and Figure 1F). Consistent with these observations, we noticed that cTregs from HIV-TB patients depicted lesser cTreg^N^ proportions compared to both HIV-LTB and HD and higher percentages of cTreg^EM^ cells compared to HD (Figure S1 in Supplementary Material). These results suggest that coinfection could induce a bias of both uTregs and cTregs to a more differentiated effector profile, leading to a greater suppression activity as shown by others for Treg^EM^ from HD (13).

### uTreg Expansion Is Related to Inflammation and CD4^+^ T Cell Differentiation Status

**Figure 2:**
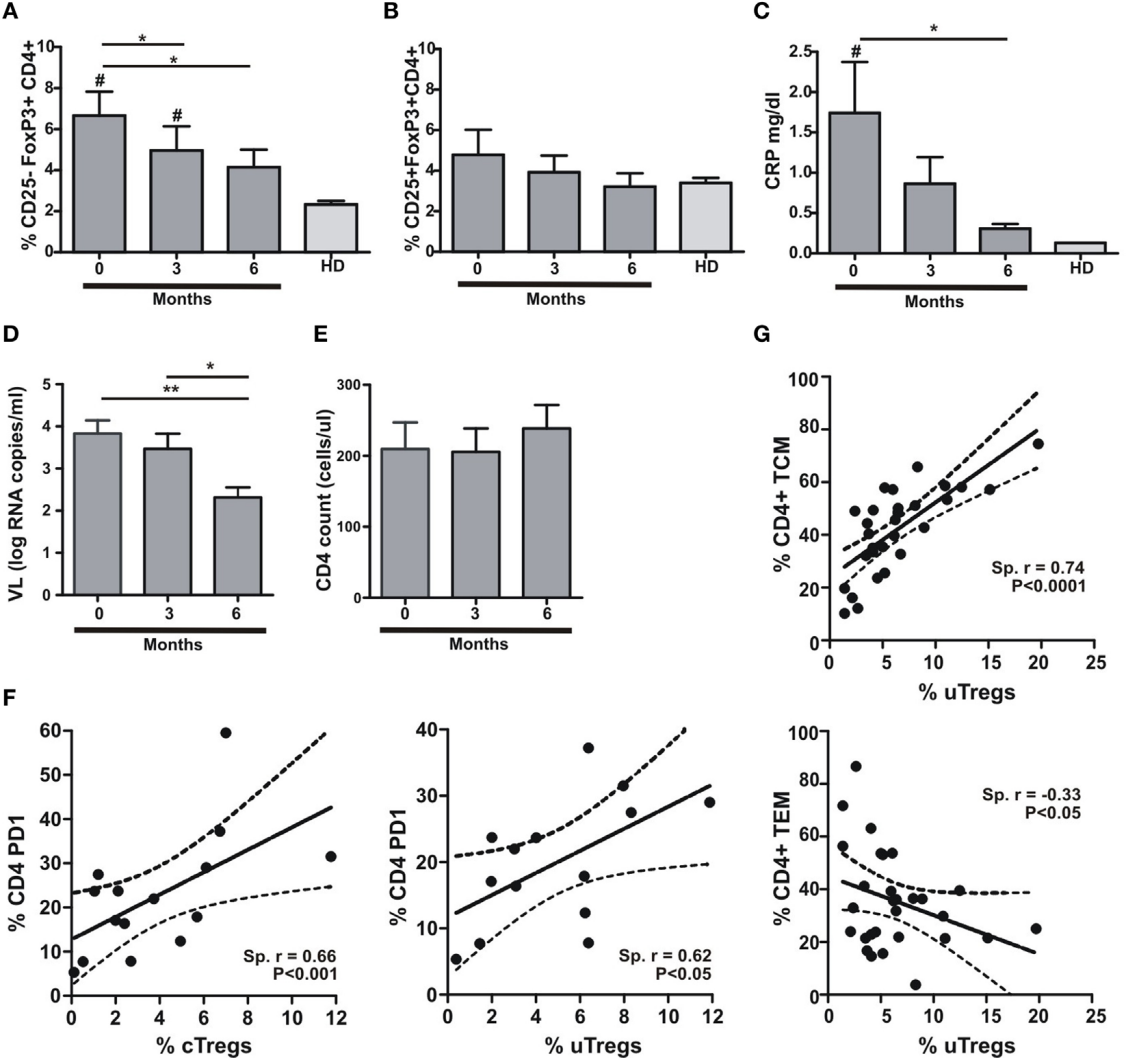
**Anti-TB treatment induces changes in uTreg but not in cTreg populations in HIV-TB patients**. **(A)** Evaluation of uTreg (CD4^+^CD25^–^FoxP3^+^), **(B)** cTreg (CD4^+^CD25^+^FoxP3^+^) frequencies by flow cytometry, **(C)** C-reactive protein (CRP) plasma concentrations, **(D)** CD4^+^ lymphocytes count, and **(E)** HIV RNA copies per milliliter in peripheral blood from HIV-TB individuals along anti-TB treatment (0, 3, and 6 months); values obtained from healthy donors (HD) were used as controls. Friedman test for paired samples was used for comparisons between consecutive visits for each population, cTreg and uTreg (**p < 0.01; *p < 0.05). For comparison between each visit and values in HD group, Mann–Whitney test was performed (^#^*p* < 0.05 was consider significant). **(F)** Correlation analysis between the % of cTreg and the % of PD1^+^CD4^+^ T lymphocytes (left) and between the % of uTreg and the % of PD1^+^CD4 T cells (right) from HIV-TB individuals. Spearman rank test was used for the evaluation of the correlation. **(G)** Correlation analysis between the % of uTreg and the % of CD4^+^ T^CM^ lymphocytes (upper panel) and between the % of uTreg and the % of CD4^+^ T^EM^ cells (lower panel) from HIV-TB individuals. Spearman rank test was used to test correlation.

It has been shown that Treg frequency is restored to normal values after infection treatment (17). Accordingly, we assessed the proportion of uTreg and cTreg cells during TB treatment at a 3-month period intervals. In parallel, we determined plasma C-reactive protein (CRP) concentrations in the same samples in order to determine whether changes in inflammation levels could escort Treg changes after a 6-month period of antitubercular treatment. We therefore observed a gradual reduction in uTreg proportions with antitubercular treatment, reaching normal levels after 6 months (Figure 2A). On the other hand, cTregs did not depict any change during TB treatment (Figure 2B). As expected, the changes observed in uTreg percentages were accompanied by a gradual normalization of CRP plasma levels (Figure 2C). These data support the idea of TB as the cause of uTreg expansion in the context of coinfection with HIV, since this population was not expanded neither in HIV^+^ nor TB individuals (data not shown). As the reduction in HIV RNA plasma viral load (VL) accompanied the normalization of uTreg proportions (Figure 2D) without CD4 count changes along treatment in our cohort (Figure 2E), it could be hypothesized that VL and its associated immune activation are driving the expansion of this subset. In this regard, we did not find any differences between groups when analyzing VL (Table 1), and also we did not observe any correlation between VL and uTregs percentages (Spearman *r*: 0.2497, *p* = 0.2091, *n* = 27 for HIV-TB individuals) suggesting that HIV-associated inflammation is not the driving force of uTreg expansion in HIV-TB coinfected patients. By contrast, when analyzing Treg proportions relative to CD4^+^ T cell activation (assessed as surface PD1 expression) we could observe a positive correlation between both uTregs and cTregs and PD1^+^CD4^+^ T cells from HIV-TB patients (Figure 2F). Also the maturation status of CD4^+^ T cells correlated with uTreg proportions, since CM CD4^+^ T cells positively correlated with uTreg proportions, whereas EM CD4^+^ T cells negatively correlated with uTregs in HIV-TB individuals (Figure 2G), but not in HIV^+^ or HIV-LTB individuals (data not shown). Overall, these data suggest that uTreg expansion is associated with lymphocyte activation and CM differentiation of CD4 T cells in HIV-TB coinfected individuals, whereas this expansion is independent of HIV RNA plasma VL.

### Phenotypic Characterization of Treg Populations in HIV-TB Individuals

**Figure 3:**
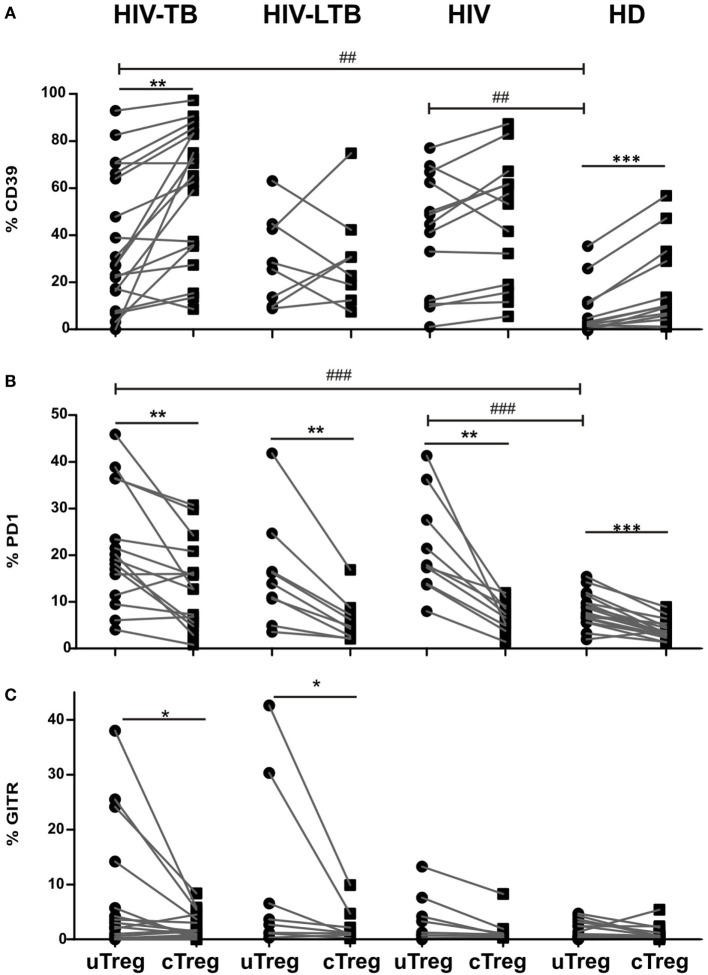
**uTreg differ in CD39, programmed cell death protein 1 (PD1) and glucocorticoid-induced tumor necrosis factor receptor (GITR) expression levels with cTreg population**. Comparison of the **(A)** CD39, **(B)** PD1, and **(C)** GITR expression between uTreg and cTreg from HIV-TB, HIV-LTB, HIV, and healthy donors (HD) individuals. Wilcoxon rank test was used for paired statistical analysis. For the comparison between the four groups, Kruskal–Wallis test was performed followed by Dunns posttest. *p < 0.05; **p < 0.01; ***p < 0.001; ^##^*p* < 0.01; ^###^*p* < 0.001.

Previously, other authors have hypothesized that CD25^−^FoxP3^+^CD4^+^ T cell population in SLE could encompass Tregs without CD25 expression or, contrary, activated effector T cells with a transient FoxP3 expression (15). To address this issue, we performed a comparative phenotypic analysis between cTregs and uTregs by evaluating the *ex vivo* expression of the surface molecules CD39, PD1, GITR, and CD127, since they had been associated with regulatory function in Tregs (3, 8, 13, 18, 19). An increase in CD39 expression in HIV-TB and HIV^+^ individuals was observed compared to HD for both uTreg (Figure 3A) and cTreg (Figure S1 in Supplementary Material). Accordingly, PD1 proportions increased in peripheral uTreg from HIV-TB and HIV patients compared to HD (Figure 3B) and also in cTreg from HIV-TB compared to HD (Figure S1 in Supplementary Material). Finally, GITR expression in uTregs did not differ between groups (Figure 3C) but HIV-TB patients depicted higher frequencies of GITR^+^ cTregs compared to HIV^+^ and HD individuals (Figure S1 in Supplementary Material). Continuing our analysis, we observed that CD39 expression was diminished on uTregs compared to cTregs both in HIV-TB and HD groups (*p* < 0.01, Figure 3A). Interestingly, PD1 expression was increased on uTregs compared to cTregs from all study groups (*p* < 0.05, Figure 3B), therefore suggesting an exhausted phenotype for uTregs irrespective of the individual’s condition. When analyzing GITR expression, we observed significantly increased proportions of this molecule in uTregs compared to cTregs from the same individuals in both HIV-TB and HIV-LTB (*p* < 0.05, Figure 3C), suggesting a *Mtb*-driven modulation of this molecule. By contrast, we did not observe any difference on CD127 expression between Treg populations (Figure S3 in Supplementary Material). Overall, these results indicate that HIV-TB coinfection induces phenotypical changes and a diminished regulatory capacity of uTregs compared to cTregs based on the lower expression of CD39 and higher levels of PD1, the latter usually associated with an exhausted phenotype (20).

**Figure 4:**
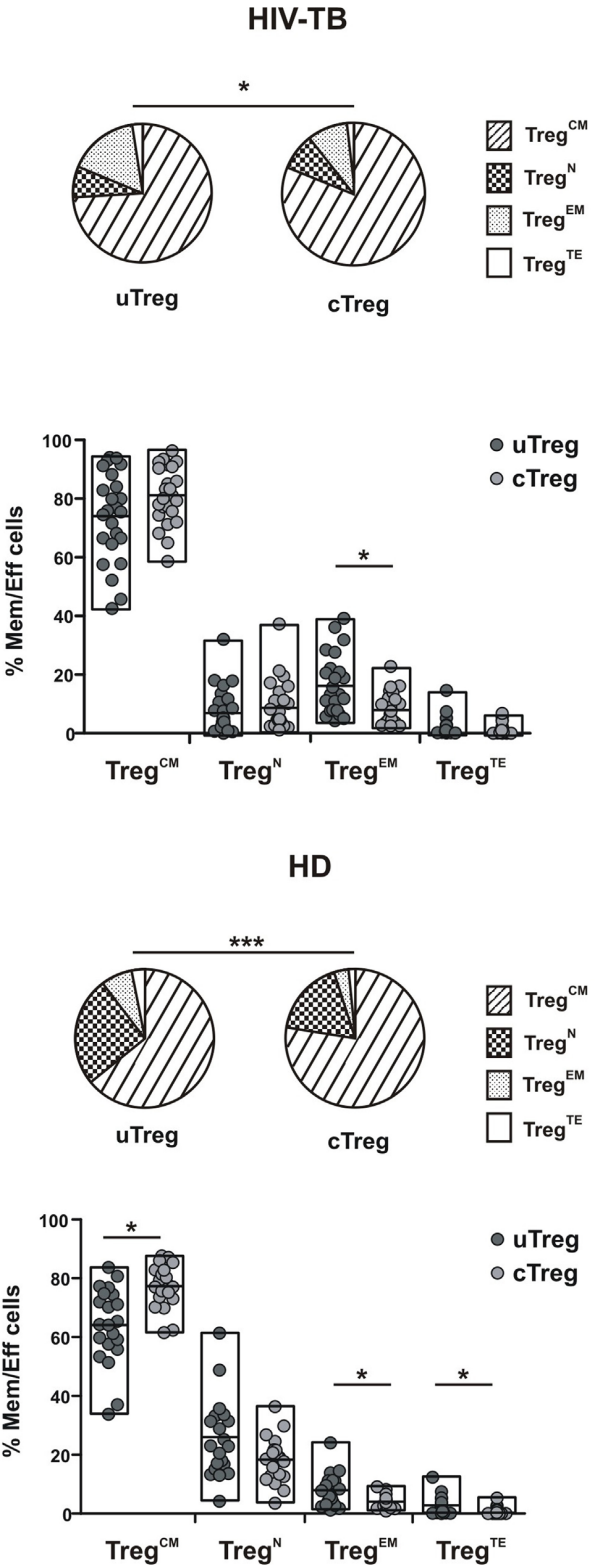
**Differing maturation status of uTreg and cTreg in HIV-TB and healthy donors (HD) individuals**. Evaluation of effector/memory phenotype distribution between uTreg and cTreg in HIV-TB (upper panel) and healthy donors (HD) (lower panel) individuals. Pie charts summarize the data and each slice corresponds to the mean proportion of uTreg or cTreg cells for each phenotype. Each point represents a single individual. Comparisons between phenotype distributions were performed using the partial permutation test follow by Kruskal–Wallis test and the Dunn’s multiple comparisons posttest. Asterisks indicate significant difference between groups. *p < 0.05; **p < 0.01; ***p < 0.001.

We then compared the memory/effector phenotype between uTreg and cTreg populations in the four study groups, based on the surface expression of CD27 and CD45RA as stated above. Partial permutation test evinced differences between memory/effector distributions between uTreg and cTreg in HIV-TB, HD, and HIV-LTB individuals (Figure 4; Figure S2 in Supplementary Material, pies), due to increased proportions of EM uTreg in the infected groups. Additionally, increased percentages of terminal effectors and diminished proportions of CM were observed in uTreg compared to cTreg in HD (Figure 4). Finally, HIV individuals showed similar memory/effector phenotypes between uTreg and cTreg populations (Figure S2 in Supplementary Material). These data suggest a more differentiated phenotype for uTregs than cTregs, irrespective of the individuals’ infection status.

**Figure 5:**
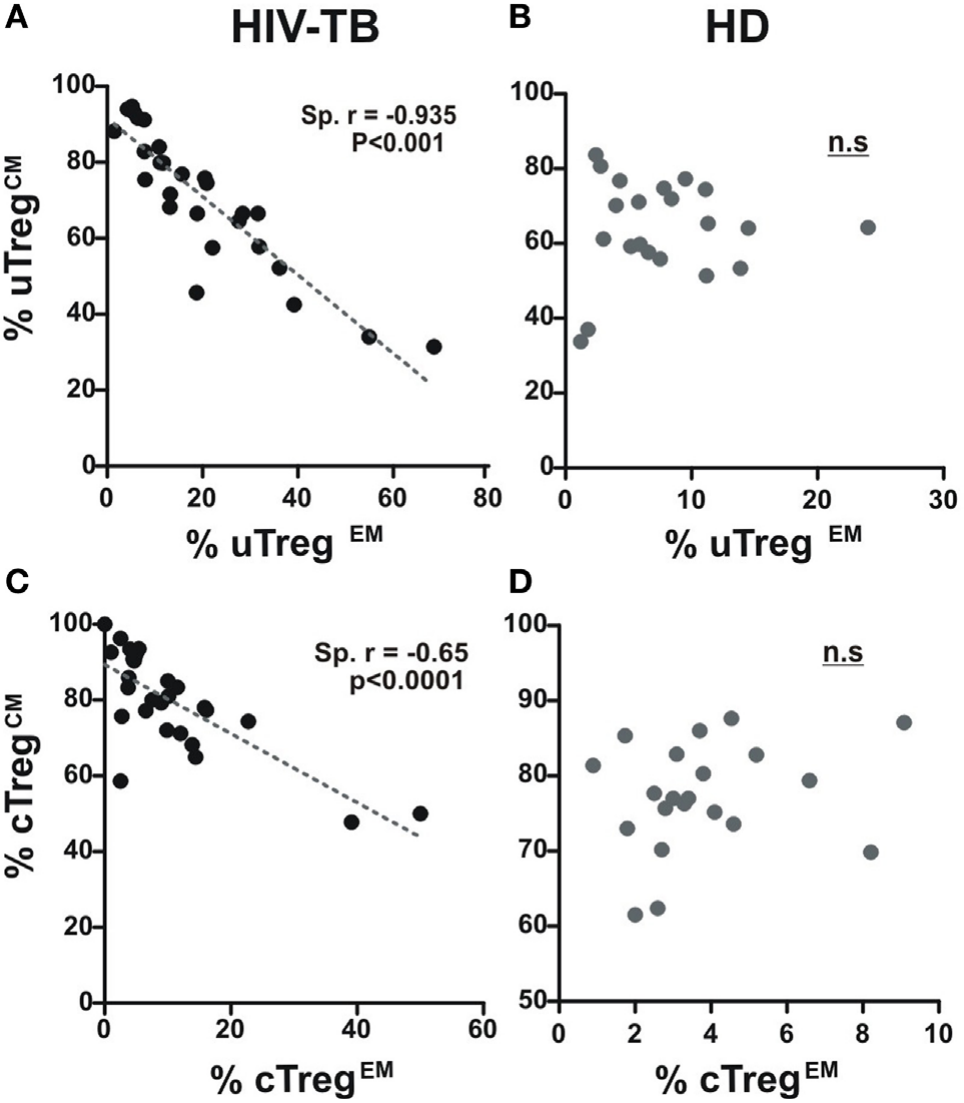
**A distinct effector/memory differentiation path occurs in Treg lymphocytes from coinfected patients**. Correlation analysis between the % of uTreg^CM^ and the % of uTreg^EM^ and between the % of cTreg^CM^ and the % of cTreg^EM^ from **(A,C)** HIV-TB and **(B,D)** healthy donors (HD) individuals in order to infer recent effector/memory transitions as described previously (13). Spearman rank test was used for the evaluation of the correlation. *p* < 0.05 was considered significant.

Differences in circulating effector/memory subset proportions may occur due to variations in their differentiation path (13). We therefore evaluated transitions between effector and memory populations by analyzing correlations between subset proportions as described previously (13, 21). Thus, a negative correlation between the percentages of two given populations suggests that transitions between those subsets are prone to occur. By doing these analyses, we observed a strong negative correlation between CM and EM subsets proportions for both uTreg and cTreg from HIV-TB individuals (Figures 5A,C). Remarkably, these correlations were not evident for the other groups (Figures 5B,D for HD and data not shown for HIV-LTB and HIV individuals). These results suggest the occurrence of a distinct differentiation path induced by *Mtb* and/or HIV over Treg lymphocytes in coinfected individuals that may account for the increment in uTreg^EM^ and cTreg^EM^ proportions observed.

### Functional Analysis of Treg Populations in HIV-TB Individuals

**Figure 6:**
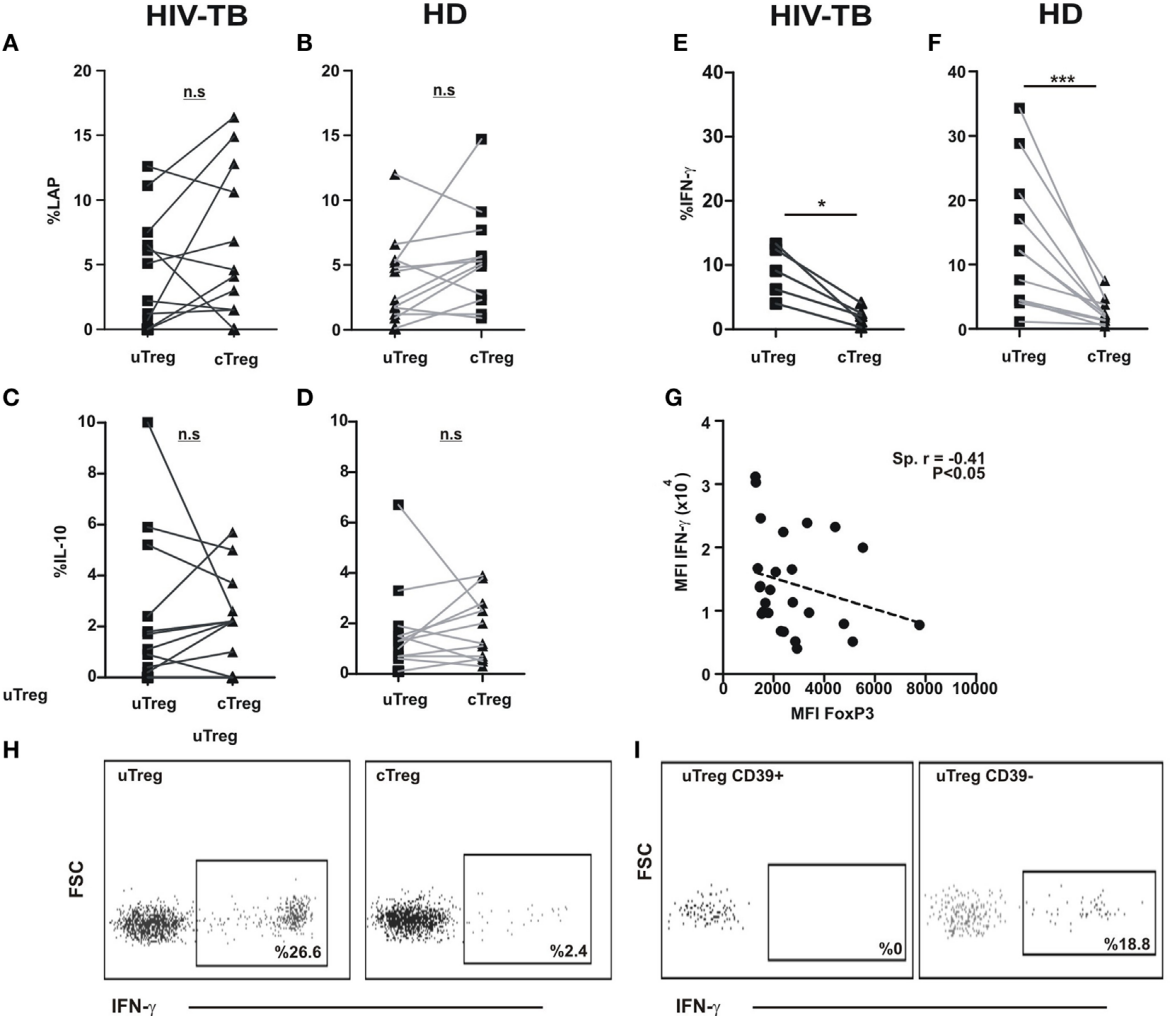
**uTregs produce a distinct pattern of cytokines and high levels of IFN-γ**. PBMC from HIV-TB and healthy donors (HD) were stimulated with PMA/Io and analyzed by flow cytometry for the indicated cytokines. Comparison of latency-associated peptide (LAP) expression between uTreg and cTreg from **(A)** HIV-TB and **(B)** HD persons. Analysis of IL-10 production between uTreg and cTreg from **(C)** HIV-TB and **(D)** HD individuals. Evaluation of IFN-γ production in the uTreg and cTreg populations from **(E)** HIV-TB and **(F)** HD. **(G)** Correlation analysis between FoxP3 and IFN-γ mean fluorescence intensity (MFI) in Tregs. Wilcoxon test was used for statistical analysis. **(H)** Representative flow cytometry dot plots depicting IFN-γ production from uTregs (left) and cTregs (right). **(I)** Representative flow cytometry graphs showing IFN-γ production from CD39^+^ uTregs (left) and CD39^−^ uTregs (right). Each point represents an individual subject. Asterisks indicate significant differences between groups. *p < 0.05; ***p < 0.001. Spearman test was performed for correlation analysis.

Human Tregs have the potential of secreting a plethora of cytokines (i.e., IL-10, TGF-β, IL-17, IFN-γ among others) (10). Therefore, we sought to investigate the functional potential of cTregs and uTregs in order to better define the nature of uTregs. We initially stimulated PBMC from HIV-TB and HD individuals in the presence of PMA and ionomycin; then we assessed IL-10 and IFN-γ production and LAP expression—a component of TGF-β prior to secretion by the cell—on Tregs by flow cytometry. We then observed a similar capacity of IL-10 secretion and LAP expression for uTregs and cTregs, both in HIV-TB and HD (Figures 6A–D,H). Also, similar levels of both cytokines and peptide expression were detected when comparing HIV-TBs cTregs versus HDs cTregs (not shown). Surprisingly, when comparing IFN-γ production between Treg populations, we detected a prominent population of IFN-γ-producing uTregs both in HIV-TB and HD groups (Figures 6E–G) that was restricted to the CD39^neg^ Treg subset (Figure 6I). Moreover, we observed a negative correlation between IFN-γ and FoxP3 expression intensity levels on a *per cell* basis (Figure 6G), according to a negative regulation of IFN-γ *loci* by the FoxP3 transcription factor as described elsewhere (22).

**Figure 7:**
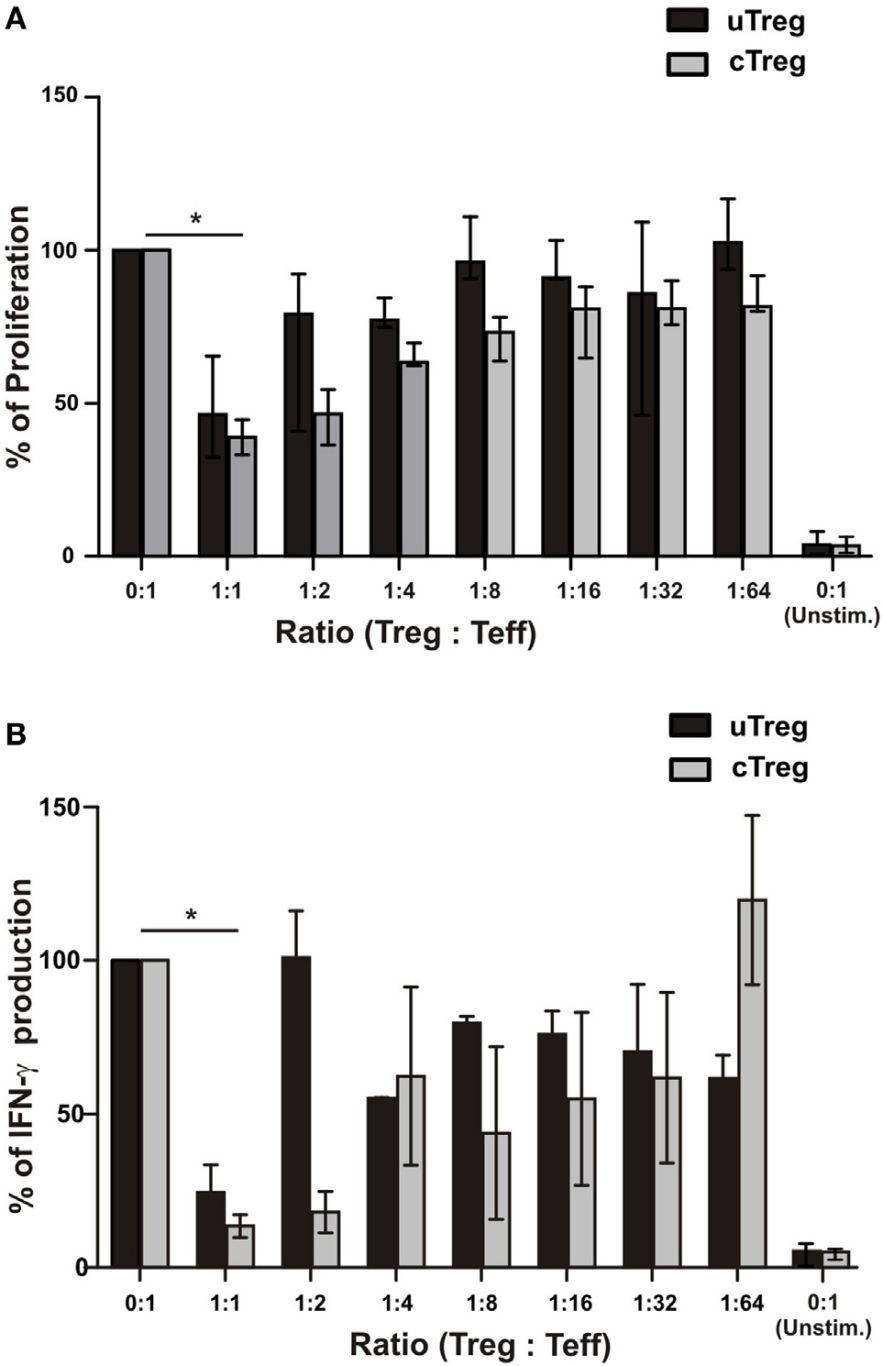
**Functional characterization of uTregs**. FACS-isolated CD4^+^CD25^−^CD39^−^ effector T cells were stimulated with anti-CD3 plus allogeneic mitomycin-treated PBMCs and cocultured with CD4^+^CD25^−^CD39^+^ uTregs or CD4^+^CD25^+^CD39^+^ cTregs cells at the indicated Treg:Teff ratios. In addition, effector T cells were left unstimulated in order to determine basal proliferation. After 5 days, proliferation and IFN-γ secretion were evaluated. Proliferation levels of stimulated CD4^+^CD25^−^CD39^−^ effector T cells without any Treg population were defined as 100%. **(A)** Proliferation of effector T cells at different Teff/Treg ratios. **(B)** IFN-γ production in cell-free supernatants from cocultures was assessed by ELISA. Bars represent mean values ^+^ SEM from three individual experiments. *p < 0.05.

Finally, in order to compare the suppressor capacity of uTreg and cTreg, we analyzed their ability to suppress lymphocyte proliferation and cytokine production *in vitro*. As we observed that CD39 expression was highly increased in both uTregs and cTregs compared to only 2% of CD25^−^FoxP3^−^CD4^+^ effector T cells expressing the inhibitory molecule (Figure S4 in Supplementary Material), we decided to use CD25^+^CD39^+^CD4^+^ as a surrogate population for regulatory T cells in the subsequent functional experiments. We thus isolated CD25^+^CD39^+^CD4^+^ T cells (cTreg), CD25^−^CD39^+^CD4^+^ (uTregs), and CD25^−^CD39^−^CD4^+^ (T-cell responder population) by FACS sorting (Figure S4 in Supplementary Material). For the assessment of their suppressor capacity, CD25^−^CD39^+^CD4^+^ or control CD25^+^CD39^+^CD4^+^ T cells from HD were cocultured with responder CD25^−^CD39^−^CD4^+^ T cells at different ratios. Therefore, it could be observed that uTregs were capable of suppressing the polyclonal-induced proliferation of responder T cells by more than 60% when cocultured at a 1:1 ratio, showing lower inhibitory capacity at higher Treg:effector ratios when compared to cTregs (Figure 7A). Moreover, uTreg and cTreg cells were analyzed for their capacity to suppress IFN-γ production from polyclonally stimulated effector T cells. Thus, as observed for the proliferative cell’s competence, a significant suppression of cytokine production capacity was achieved by both uTregs and cTregs from HD (Figure 7B). Notably, although only near 50% of CD25^−^CD39^+^CD4^+^ T cells were Foxp3^+^, their suppressor capacity was similar to that appreciated on CD25^+^CD39^+^CD4^+^ Tregs, which depicted 90% of Foxp3 expression (Figure S4 in Supplementary Material). These data strongly indicate that “unconventional” Tregs are a Treg population with preserved inhibitory capacity, albeit uTregs display a distinct phenotype with higher expression of surface CD39 and PD1 and a more differentiated memory/effector profile.

## Discussion

*Mycobacterium tuberculosis* and HIV coinfection entails a scenario where both infections worsen the outcome of each other, leading to the progression of both diseases. The relevance of Tregs in this context is a matter of continuous study, since Tregs are involved in both TB and HIV immune responses (6, 23). In TB, Tregs control immunopathology and also are able to down-modulate effector responses, necessary for pathogen eradication (24). Tregs are highly heterogeneous, with differing phenotypes besides their FoxP3 expression. Similar to effector T cells, TCR activation induces Tregs differentiation resulting in dissimilar memory populations (25). In turn, each population has its own inhibitory potential, since it has been described that EM Treg lymphocytes display a more potent inhibition capacity compared to terminal effector or CM cells (13). Moreover, surface proteins can also orchestrate Treg function, as CD39 can inhibit T cell effector functions by modulating adenosine concentration (26), PD1 can deliver inhibitory signals through PDL1 engagement to effector T lymphocytes (18), and GITR can enhance Treg function (27). Taking into account our previous observations as a starting point, we aimed to study the expanded FoxP3^+^CD25^−^CD4^+^ population in a cohort of HIV-TB coinfected individuals from Argentina. This T cell population was previously observed in the context of SLE (8, 9), raising the question whether these cells were activated effector T cells transiently expressing the transcription factor FoxP3 or were abnormally differentiated Tregs with impaired regulatory functions. We observed that this unique population depicted similarities in their memory/effector status compared to FoxP3^+^CD25^+^CD4^+^ “conventional” Tregs, but had some differences regarding CD39 and PD1 expression. Interestingly, this uTreg population exerted a huge ability to secrete IFN-γ, while it could efficiently inhibit effector T cell responses. IFN-γ-producing FoxP3^+^ Tregs were detected in the context of graft-versus-host disease, in *Listeria monocytogenes* infection in mice (28) and also in human autoimmune disease (29). This uTreg expansion could be probably induced by TB, since we observed that after TB treatment uTreg levels reached normal ranges on peripheral blood from HIV-TB patients. Furthermore, patients’ immune status was related with this expansion, since uTregs frequency negatively correlated with CD4^+^ T cell count in HIV-TB patients and positively correlated with PD1 expression in CD4^+^ T cells. These results could be related to the data showed by Amarnath and colleagues indicating a role for PD1–PDL1 axis on the differentiation of Th1 cells into regulatory T cells (30). Our results also indicate that the maturation status of T cells is related to uTreg expansion, since EM CD4^+^ T cells percentages (which are the main effector populations against the pathogen) negatively correlated with uTreg proportions, therefore suggesting a bias to a less differentiated phenotype for effector T cells in an scenario where uTregs could be inhibiting anti-mycobacterial responses.

Maturation status can define the functional capacity of a given cell population, as described for Tregs and conventional T cells (13, 31). We observed that uTreg from coinfected individuals exhibited a more differentiated phenotype with an expansion of the highly suppressor Treg^EM^ subset (13), probably resulting in diminished antitubercular immune responses at the site of infection. Our studies on the characterization of uTregs showed that these cells had phenotypic differences from cTregs, and these differences became more noticeable during HIV-TB coinfection. CD39, a Treg-associated ectonuclease, acts hydrolyzing ATP or ADP into AMP, thereby generating an anti-inflammatory environment (26). Thus, a lower expression of CD39 on the surface of uTreg (as shown in Figure 3) could be related to a defective ability to control immune responses. Furthermore, the expression of PD1, a molecule associated with T cell exhaustion in the context of chronic infections such as HIV (32) was augmented on uTreg compared to cTreg, therefore suggesting an exhausted phenotype for this population. However, a recent study from Park et al. has described an active role of PD1 (present on the surface of LTreg) in the inhibition of LT CD8^+^ responses to a chronic viral infection, where interaction with PDL1 on the surface of CD8^+^ T lymphocytes actively inhibited the effector function of these cells (18). Therefore, an increased expression of PD1 on the surface of uTreg would result in an increased inhibitory capacity, contrary to the former governing paradigm.

On the other hand, little progress has been made on the study of the effect of signaling GITR in Treg. However, it has been recently described that signaling through GITR induces an increase in the proliferation of Treg lymphocytes *in vitro* and *in vivo* (33). Therefore, intracellular signaling of GITR could be a stimulus of cellular activation, increasing the regulatory capacity of Tregs. Therefore, in view of a phenotype showing a low expression of CD39 concomitantly with high levels of PD1 and GITR on the surface of uTreg from HIV-TB patients, a possible scenario could be that this population could retain its ability to modulate T cell responses through increased expression of PD1 and GITR. Conversely, this modulating capacity would be compromised due to the low expression of CD39 and high expression of PD1, showing an exhausted phenotype for these unconventional regulatory cells. A third possibility could be that this unique population acquires novel strategies for inhibition of T cell responses, as described for LES, where CD25^−^ Tregs could inhibit T cell proliferation but not IFN-γ production (8). We addressed this question by studying the inhibitory capacity of uTregs in an *in vitro* setting and found that uTreg cells have the ability to suppress proliferation and IFN-γ production of effector lymphocytes to an extent comparable to cTreg (Figure 7).

The ability to modulate the responses from LTreg involves several mechanisms, such as the secretion of modulatory cytokines like IL-10 or TGF-β (34–36). Our results show a similar ability to produce and secrete IL-10 and TGF-β by conventional and unconventional Treg populations, therefore indicating that their modulatory capacity through soluble mediators would be comparable. Remarkably, uTregs from coinfected patients depicted high levels of IFN-γ production compared to cTregs. This ability for IFN-γ secretion by CD4^+^CD25^−^FoxP3^+^ cells was previously shown by Yang in the context of lupus patients (9). Nevertheless, the levels of IFN-γ observed by these authors were much lesser than our observations perhaps due to the gating strategy they used based on CD127 expression (9). These authors also analyzed the ability of uTreg cells to inhibit effector T lymphocytes responses. Unfortunately, they used CD127 as a surrogate marker for Treg isolation, obtaining a CD4^+^CD25^−^CD127 low/neg population with a very low proportion of FoxP3 [around 9% (9)], therefore achieving inconclusive results. On the other hand, Bonelli obtained around 53% of CD4^+^CD25^−^CD127^−^ T cells, which inhibited T cell proliferation but not IFN-γ production (8). A possible explanation for this finding is that Bonelli’s Tregs could encompass IFN-γ producers, a population that was not investigated in that work (8). Thus, Yang’s and Bonelli’s works provide evidence supporting the existence of a regulatory subpopulation with the capacity for IFN-γ production which was not observed in other scenarios beyond SLE or in the present study focused on HIV-TB coinfection.

Finally, we provide evidence regarding the pathogenesis of TB in the context of HIV infection, describing a unique population of regulatory T cells which may arise from a preexisting pool that is expanded under the inflammatory milieu generated by coinfection. This population, described previously only during another inflammatory disease such as autoimmune LES, may be involved in the pathogenic mechanisms developed during HIV-TB coinfection. Our results show for the first time an in-depth analysis of unconventional CD25^−^ Tregs that may provide significant information in order to generate novel approaches for a better management of *Mtb* infection in patients coinfected with HIV.

## Ethics Statement

The Ethics Committees from Fundación Huésped and from the University of Buenos Aires School of Medicine approved the current study. In addition, written informed consent was documented from all study subjects.

## Author Contributions

MA designed and performed the experiments, analyzed data, and wrote the manuscript; GS and MV performed some experiments and analyzed data; NL, DA, GB, HP, OS, and HS recruited the patients and analyzed data; and MQ designed the experiments, analyzed data, and wrote the manuscript.

## Conflict of Interest Statement

The authors declare that the research was conducted in the absence of any commercial or financial relationships that could be construed as a potential conflict of interest.

## Abbreviations

TB, tuberculosis; LTB, latent TB infection; uTreg, unconventional regulatory T lymphocyte; cTreg, conventional regulatory T lymphocyte.
