# Supplementary material for: Expansion of CD25-Negative Forkhead Box P3-Positive T Cells during HIV and Mycobacterium tuberculosis Infection
Source: Front Immunol. 2017 May 9;8:528. doi: 10.3389/fimmu.2017.00528 (PMC5422469; doi:10.3389/fimmu.2017.00528)
Supplement: Supplementary file 1 [file Data_Sheet_1.PDF]

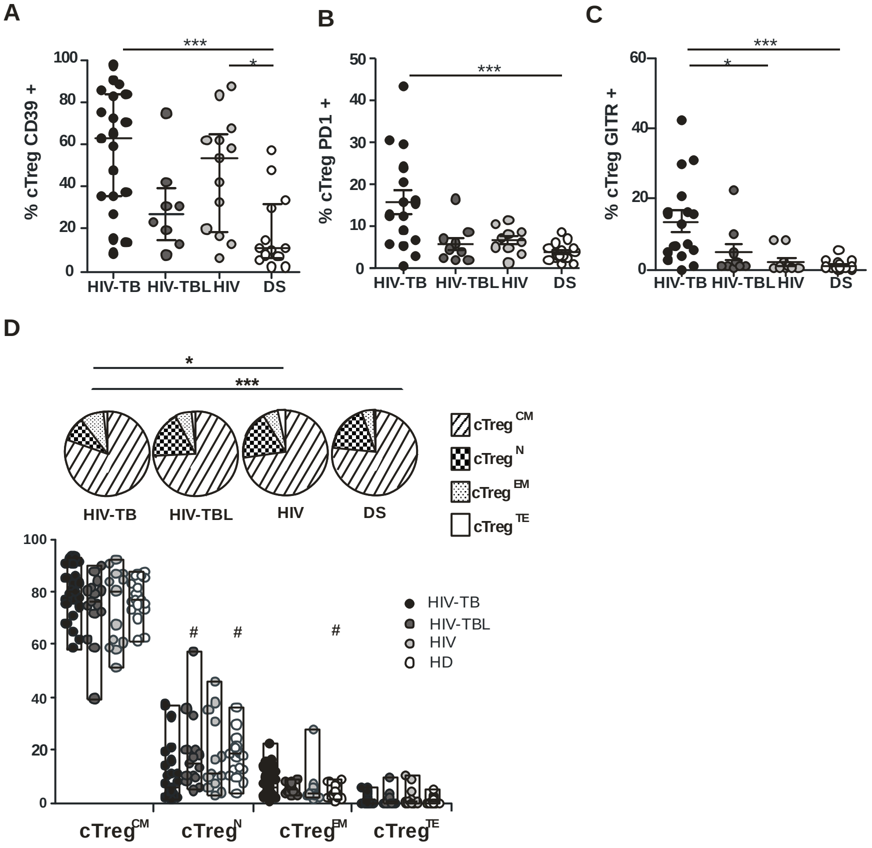

Supplementary Figure 1. *Analysis of cTreg population across the spectrum of HIV-TB infection.* PBMC from HIV-TB, HIV-LTB, HIV+ and HD individuals were stained for cell surface expression of CD4, CD25, CD39, PD1, GITR, CD45RA, CD27 and intracellular FoxP3 and analyzed by flow cytometry. Cells were gated based first on the basis of CD4 expression, then the CD4+ cells were analyzed for the expression of CD25 and FoxP3 in order to define “conventional” and “unconventional” Tregs, and finally the expression of CD39, PD1, GITR or CD45RA/CD27 was determined in cTregs. Proportions of A) CD39+ B) PD1 and C) GITR on uTregs between the four studied groups. D) The expression of CD27 and CD45RA on CD4+T cells from HIV-TB, HIV-LTB, , HIV+, and HD individuals was analyzed by flow cytometry; (top) Pie charts summarize the data and each slice corresponds to the mean proportion of cTreg cells for each phenotype. (Bottom) Possible phenotypes are shown on the *x*-axis whereas percentages of distinct T-cell subsets within

cTreg cells are shown on the *y*-axis. For the comparison between the four goups Kruskall Wallis was performed, followed by Dunns post-test. Horizontal lines represent the median range and each point represents an individual subject; asterisks indicate a significant difference between groups;*: $p < 0.05$; ***: p <, 0.001. Comparisons of phenotype distribution were performed using the partial permutation test as described in (36) and the Kruskall–Wallis test followed by Dunn's multiple comparisons posttest.

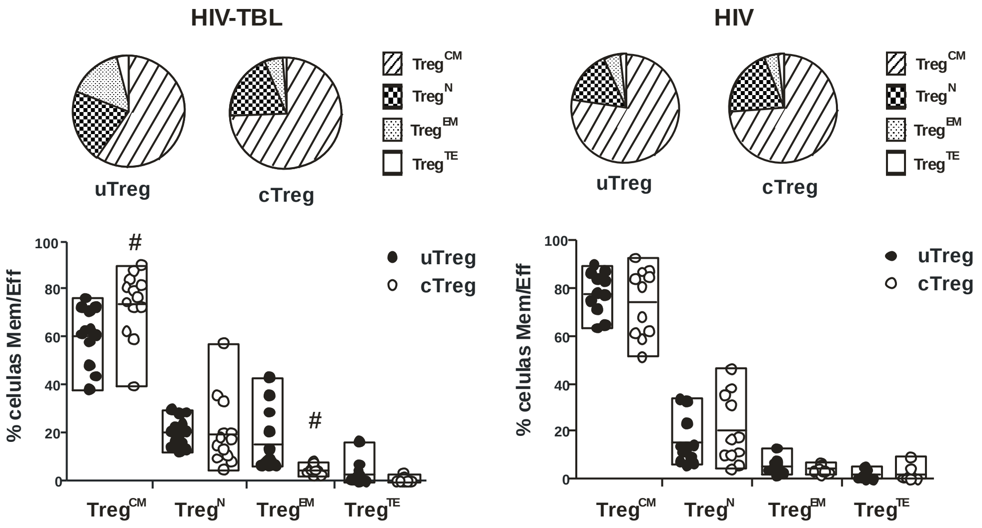

Supplementary Figure 2. *Analysis of maturation status of uTreg and cTreg in HIV-TBL and HIV individuals*. Evaluation of Effector / Memory phenotype distribution between uTreg and cTreg in HIV-TBL (left panel) and HIV (right panel) individuals. Pie charts summarize the data and each slice corresponds to the mean proportion of uTreg or cTrteg cells for each phenotype. Each point represents a single individual. Comparisons between phenotype distributions were performed using the partial permutation test follow by Kruskall–Wallis test and the Dunn's multiple comparisons posttest. Hash marks indicate a significant difference between groups. #: $p<0.05$.

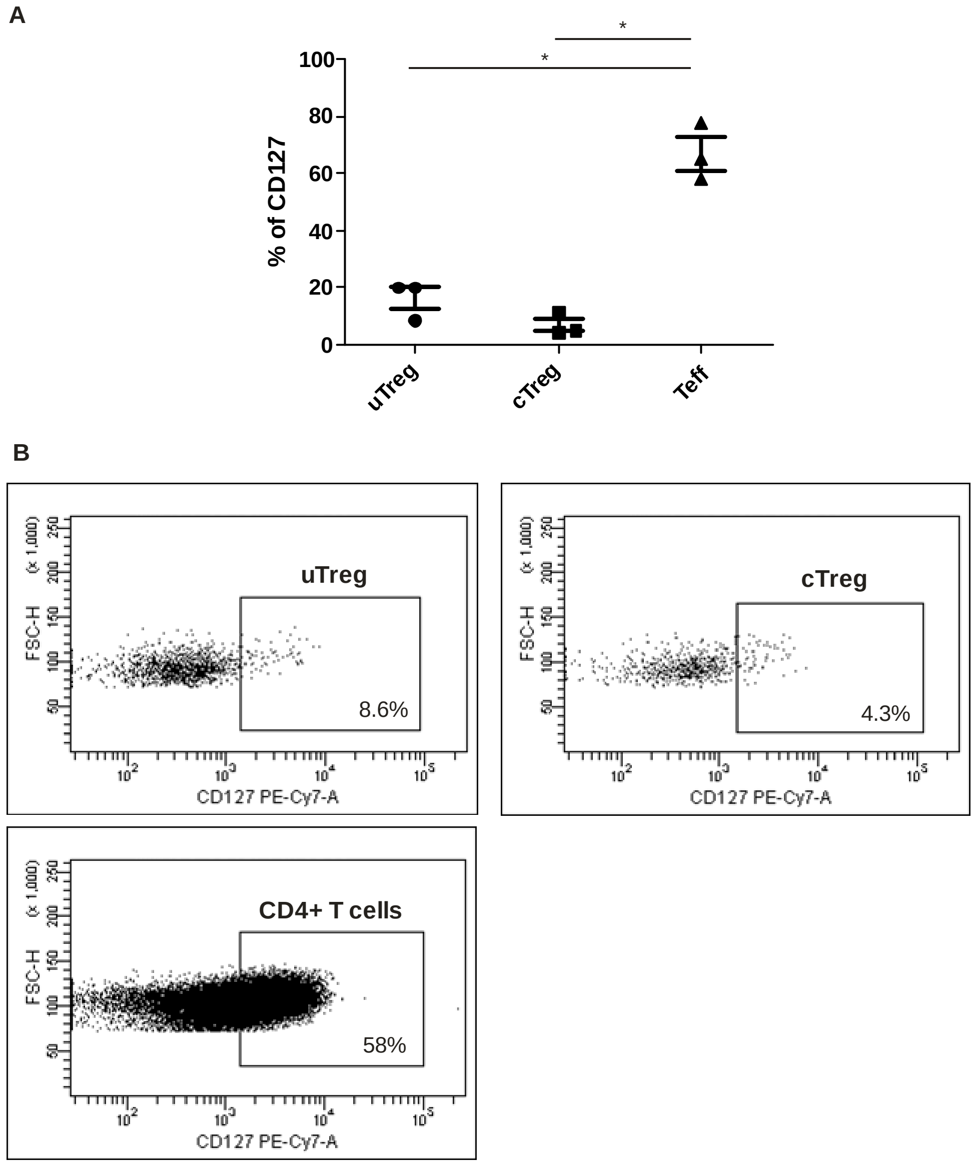

Supplementary Figure 3. *Analysis of CD127 expression on Treg populations in HIV-TB infection.* PBMC from HIV-TB individuals were stained for cell surface expression of CD4, CD25, CD127 and intracellular FoxP3 and analyzed by flow cytometry. Cells were gated based first on the basis of CD4 expression, then the CD4+ cells were analyzed for the expression of CD25 and FoxP3 in order to define “conventional” and “unconventional” Tregs, and finally the expression of CD127 was determined in both Tregs and effector T

cells (Teff). A. Comparison of CD127 expression between uTregs, cTregs and effector T cells. For the comparison between the three goups Kruskall Wallis was performed, followed by Dunns post-test. Horizontal lines represent the median range and SD and each point represents an individual subject; asterisks indicate a significant difference between groups; ***: p <, 0.001. B) Gating strategy used to isolate CD4+ CD25+ CD39+ (cTregs) and CD4+ CD25- CD39+ (uTregs)(left panel) and the resulting FoxP3 expression from the obtained fractions. A representative experiment out of three is shown.

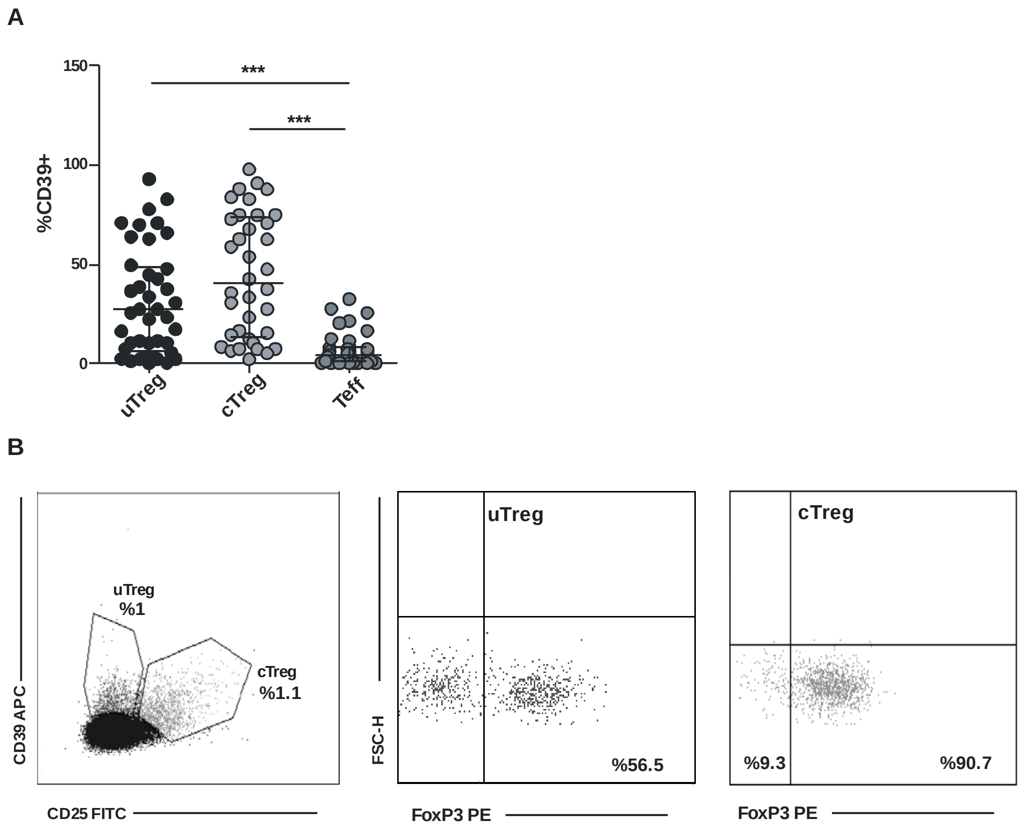

Supplementary Figure 4. *Use of CD39 as a putative marker for isolation of uTregs.* A) Comparison of CD39 expression between uTregs, cTregs and effector T cells. For the comparison between the three goups Kruskall Wallis was performed, followed by Dunns post-test. Horizontal lines represent the median range and SD and each point represents an individual subject; asterisks indicate a significant difference between groups; ***: p <, 0.001. B) Gating strategy used to isolate CD4+ CD25+ CD39+ (cTregs) and CD4+ CD25- CD39+ (uTregs)(left panel) and the resulting FoxP3 expression from the obtained fractions. A representative experiment out of three is shown.
